# Genetic Population Structure and Distribution of the Small Giant Clam *Tridacna maxima* in Indo‐Pacific Coral Reefs: History Dynamics, Present Status and Future Trends

**DOI:** 10.1002/ece3.71965

**Published:** 2025-08-08

**Authors:** Haojun Chi, Zhongli Sha, Lin He, Min Hui

**Affiliations:** ^1^ Laboratory of Marine Organism Taxonomy and Phylogeny, Qingdao Key Laboratory of Marine Biodiversity and Conservation Institute of Oceanology, Chinese Academy of Sciences Qingdao China; ^2^ University of Chinese Academy of Sciences Beijing China; ^3^ Laboratory for Marine Biology and Biotechnology, Qingdao Marine Science and Technology Center Qingdao China; ^4^ Shandong Province Key Laboratory of Experimental Marine Biology Institute of Oceanology, Chinese Academy of Sciences Qingdao China

**Keywords:** climate change, conservation, coral triangle, giant clam, population genetics

## Abstract

Various hypotheses have been proposed to explain the origin of the high biodiversity in the Indo‐Malay Archipelago (IMA), such as the center of origin, overlap zone, and accumulation center; yet these theories remain subjects of ongoing debate. The small giant clams, *Tridacna maxima*, are iconic inhabitants of coral reefs and are widely distributed in the Indo‐West Pacific. However, due to overexploitation and climate change, wild populations of most giant clam species worldwide have been severely impacted and are now endangered. In this study, *cytochrome c oxidase I* (*COI*) gene sequences of 35 *T. maxima* individuals from two populations in the South China Sea (SCS) were amplified and sequenced. These data were integrated with published *COI* sequences of *T. maxima* from other studies. A total of 610 individuals in 34 locations across the Indian Ocean to the Central Pacific were included in the population genetic analysis based on a 417 bp fragment of *COI*. The genetic differentiation index *Φ*_st_ = 0.856 (*p* = 0.000) shows a significantly differentiated genetic structure, which can be categorized into six distinct groups from west to east, as previously suggested. The populations in the SCS exhibited strong connectivity with the IMA populations, forming a single group. Gene‐flow analysis revealed a pattern of migration from the Seas of Southeastern Asia (SEA) towards both the eastern and western directions, supporting the center of origin hypothesis for the high biodiversity of IMA. Historical population dynamics analysis indicated that most groups experienced expansion, primarily associated with the late Pleistocene glaciations. Moreover, the Species Distribution Model (SDM) predicted that climate change might lead to a significant reduction in suitable habitats for *T. maxima* and a slight shift towards higher latitudes. These results are expected to provide insights into the origin of the biodiversity in the IMA and baseline data for the conservation of giant clams.

## Graphical abstract

Six distinct groups were identified in *T. maxima* across the Indo‐Pacific region, which was shaped by the ocean currents and the late Pleistocene glaciations. Gene‐flow analysis supported the center of origin hypothesis for the high biodiversity of the Indo‐Malay Archipelago. The Species Distribution Model predicted that climate change will lead to a significant reduction in suitable habitats for *T. maxima* .

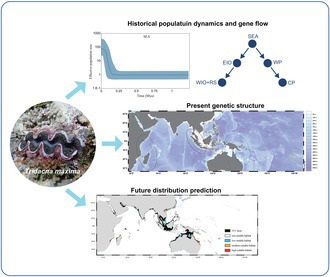

## 1 Introduction

The tropical Indo‐West Pacific (IWP) is the most biologically diverse marine region worldwide, with several distinct provinces (Briggs and Bowen 2012). Known as the Coral Triangle (CT), the IMA, located within the IWP and connecting the Philippines, Sumatra, and New Guinea, comprises a myriad of islands and peninsulas of varying sizes and geological origins (Hall 1996). This topographically intricate region fosters complex geographic structures and oceanographic current patterns (Wyrtki 1961) and is recognized as a global hotspot for marine shallow‐water biodiversity (Briggs 2000; Allen and Werner 2002). The biodiversity of the CT is shaped by a variety of factors, such as the abundance of coral reefs with high primary productivity, which provide rich resources and complex habitats in support of vast marine diversity (Adams et al. 2020). Several hypotheses have been proposed to explain the evolution of the exceptional biodiversity within the CT, such as the center of origin, the center of overlap, the center of accumulation, and the center of survival (Briggs 1999; Woodland 1983; Jokiel and Martinelli 1992; Bellwood and Meyer 2009; Bellwood et al. 2012). A recent study suggests that the high diversity of reef corals in the CT largely stems from range expansions into this region of species that initially evolved elsewhere (Huang et al. 2018). Since these explanations are not mutually exclusive, no single hypothesis may be sufficient to encapsulate the entire spectrum of its diversity (Hoeksema 2007; Barber and Meyer 2015). Hence, instead of seeking a unified hypothesis to account for biodiversity across the entire range of organisms in the CT, it might be more enlightening to inquire whether a specific hypothesis is more plausible in explaining the biogeographical distribution for a particular set of organisms with certain ecological and life history traits, which will ultimately enhance the understanding of the elements contributing to the species richness in the CT (Tornabene et al. 2015).

Giant clams (Cardiidae: Tridacninae) are emblematic mollusks of the Indo‐Pacific coral reefs, extending from East Africa to the Central Pacific (CP). Known for their substantial size, distinctive shell architecture, and vibrant mantle, giant clams play crucial roles in ecological processes of coral reef ecosystems. They provide habitat and shelter for various marine organisms and contribute carbonate through their aragonite shell formation, while their most vital role is enhancing reef productivity via high biomass production and nutrient cycling with symbiotic zooxanthellae (Neo et al. 2015, 2017). Recently, the complete genomes of four tridacnid species have been sequenced, unveiling genomic characters driven by symbiosis and historical population changes tied to geoclimatic events (Li et al. 2023, 2024a, 2024b, 2025). It has been supposed that the CT represents the contemporary epicenter of biodiversity for giant clam species (Lucas 1988). Further population genetic structure and gene‐flow studies within the distribution range of giant clams are expected to benefit understanding the mechanisms underlying the formation of high biodiversity in the CT. Current research on population genetics and molecular phylogenetics of giant clams has revealed generally well‐defined population structure and varying levels of genetic connectivity among different biogeographic populations (Huelsken et al. 2013; Hui, Kraemer, et al. 2016; Keyse et al. 2018; Neo et al. 2018; Fauvelot et al. 2020). The detection of concordant population genetic patterns in giant clams using both *COI* and nuclear microsatellites demonstrates that *COI* serves as an effective marker for genetic structure analysis in these species (Deboer et al. 2014; Hui, Nuryanto, and Kochzius 2016). This finding is consistent with studies in anemonefish (Timm et al. 2012; Dohna et al. 2015). However, previous research on giant clams has not encompassed populations from the north of the SEA, such as the SCS, lacking a comprehensive analysis that integrates all biogeographic populations. The historical dynamics and gene flow of these populations also remain unclear, hindering complete understanding of the origin of biodiversity in giant clams.

Since the 1980s, the significant rise in ocean temperatures caused by anthropogenic climate change has emerged as a primary threat to nearly all ecosystems in the contemporary world; since 1993, the rate of ocean warming has doubled (Pörtner et al. 2019). Coral reef ecosystems are highly sensitive to climate change (Heron et al. 2016), with severe heatwaves triggering widespread coral bleaching events due to the loss of zooxanthellae (Hughes et al. 2018). Giant clams also heavily rely on the photosynthesis of symbiotic algae to meet their energy demands and are vulnerable to the effects of rising sea surface temperatures induced by global warming (Andréfouët et al. 2013; Van Wynsberge and Andréfouët 2017; Van Wynsberge et al. 2018). Higher temperatures induce various physiological responses in giant clams, such as changes in respiration rates (Blidberg et al. 2000), enzyme activity levels (Zhou et al. 2019), and photosynthetic yield of the symbionts (Brahmi et al. 2021). Additionally, giant clams exhibit bleaching as a stress response to elevated temperatures and intense light exposure (Buck et al. 2002). Nowadays, wild stocks of most giant clam species are depleted worldwide, with some species locally extinct in several countries (Wells 1997; Van Wynsberge et al. 2016; Neo and Low 2018). In response to their vulnerable status, one giant clam species is classified as Critically Endangered, and three species are listed as Endangered in the International Union for Conservation of Nature (IUCN) Red List of Threatened Species (Version 2024‐2, https://www.iucnredlist.org), and all species are listed in Appendix II of the Convention for International Trade in Endangered Species of Wild Fauna and Flora (CITES) (Ramah et al. 2019). Expanding the global protected areas for giant clams is crucial for the conservation of these endangered species. Therefore, it is essential to determine the climate‐driven shifts in the distribution of giant clams to facilitate the creation of protected areas. In recent years, the species distribution model (SDM) has been increasingly applied to delineate suitable habitats for species and to predict the impacts of future climate change on species distributions, thereby providing a scientific basis for conservation planning, such as in the endangered cetaceans, corals, and many other invertebrates (De Oliveira et al. 2019; Melo‐Merino et al. 2020; Putra and Mustika 2021; Xu et al. 2022).

With the significant accumulation of available *COI* data for giant clams, *COI* becomes the most practical tool for conducting large‐scale population genetic analyses. In this study, we conduct a comprehensive population genetic analysis on the small giant clam *T. maxima* across its entire distribution range using *COI*, examine gene flow, and analyze population historical dynamics to elucidate the patterns and processes of lineage diversification, endeavoring to offer valuable insights into the origin of biodiversity in giant clams. Further, the potential habitat redistribution pattern of *T. maxima* under future climate change is predicted by simulating ecological niches based on current geographic distribution data of *T. maxima* and various ecological factors, which will provide a crucial foundation for the conservation of giant clams in the face of climate change.

## 2 Methods

### 2.1 Sampling

**FIGURE 1:**
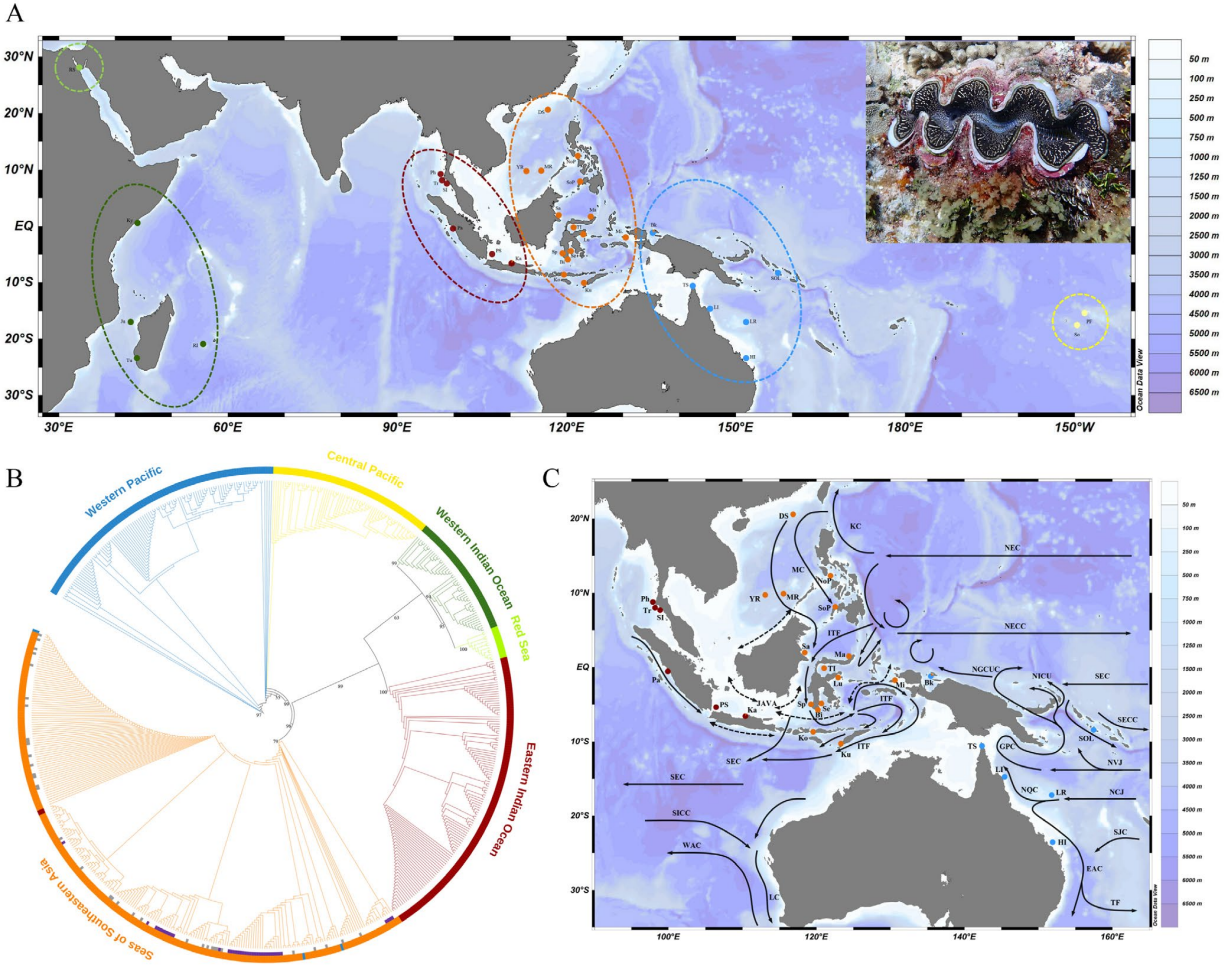
Maps showing sampling sites and genetic structure for *T. maxima* . (A) Sampling sites (for abbreviations, see Table 1) and major genetic breaks (colored circles) for *T. maxima* populations. (B) The maximum‐likelihood phylogenetic tree for giant clam individuals based on *COI* sequences. Purple branches represent individuals from the Nansha Islands, and gray branches represent individuals from the Dongsha Islands. The numbers indicate bootstrap values. (C) Surface currents with constant (solid arrows) and seasonally changing flows (dashed arrows). Kuroshio Current (KC), North Equatorial Current (NEC), Mindanao Current (MC), North Equatorial Counter Current (NECC), New Guinea Coastal Undercurrent (NGCUC), New Ireland Coastal Current (NICU), South Equatorial Current (SEC), South Equatorial Counter Current (SECC), North Vanuatu Jet (NVJ), North Caledonia Jet (NCJ), South Caledonia Jet (SCJ), North Queensland Current (NQC), Gulf of Papua Current (GPC), East Australian Current (EAC), Tasman Front (TF), Indonesian Throughflow (ITF), West Australian Current (WAC), and Southern Indian Current Circle (SICC) (Hu et al. 2015).

Mantle tissue samples of *T. maxima* were collected by SCUBA diving from the Meiji Reef and Yongshu Reef in the Nansha Islands of the SCS in 2022 and 2023 (Table 1, Figure 1A). To minimize disturbance to the clams, a biopsy method was employed, and small mantle tissues were cut underwater to ensure the survival of the clams. Tissue samples were then preserved in 96% ethanol and stored at 4°C in the laboratory.

**TABLE 1:** Summary statistics for each population of *T. maxima* .

Sites (code)	Code	Region	*n*	*N*_hp_	*h*	*π* (%)	*D*	Fs	SSD	HRI
Torres Strait	TS	WP	25	17	0.94	2.63	−1.67*	−7.94***	0.003	0.009
Lizard Island	LI	WP	22	12	0.90	2.06	−1.17	−3.73*	0.038	0.066
Lihou Reef	LR	WP	13	7	0.79	1.99	−1.40	−0.74	—	—
Heron Island	HI	WP	31	17	0.92	1.53	−1.90**	−10.68***	0.005	0.040
Solomon Islands	SOL	WP	4	4	1.00	1.35	0.65	−1.62*	—	—
Biak	Bk	WP	16	13	0.97	7.12	0.09	−1.87	—	—
Tulear	Tu	WIO	31	19	0.90	3.39	−0.69	−6.40*	—	—
Reunion Island	RI	WIO	4	3	0.83	1.73	−0.81	0.73	0.328*	0.972
Juan de Nova	Ju	WIO	6	4	0.80	1.11	−0.66	−0.56	0.037	0.138
Kenya	Ky	WIO	9	7	0.92	4.66	0.75	−0.17	0.047	0.035
Red Sea	RS	RS	13	10	0.95	1.57	−0.94	−5.61***	—	—
Yongshu Reef	YR	SEA	19	10	0.78	0.84	−2.10**	−6.59***	0.001	0.035
Meiji Reef	MR	SEA	16	8	0.76	0.88	−1.61*	−3.91**	—	—
Dongsha Islands	DS	SEA	39	22	0.85	2.28	−2.02**	−12.23***	0.008	0.021
South of Philippines	SoP	SEA	11	11	1.00	2.00	−2.11**	−8.95***	—	—
North of Philippines	NoP	SEA	17	17	1.00	2.55	−1.97**	−16.02***	—	—
Komodo	Ko	SEA	12	4	0.56	0.37	−1.18	−1.59*	—	—
Kupang	Ku	SEA	14	10	0.89	1.94	−1.37	−4.09*	—	—
Spermonde	Sp	SEA	21	10	0.69	1.29	−2.24**	−3.85*	0.007	0.028
Bira	Bi	SEA	10	9	0.98	1.82	−1.86*	−5.32***	—	—
Sembilan Islands	Se	SEA	12	8	0.85	1.06	−2.07**	−4.36**	0.006	0.063
Luwuk	Lu	SEA	16	9	0.86	1.01	−1.31	−4.82***	—	—
Togian Islands	TI	SEA	21	16	0.96	1.73	−2.18**	−12.00***	—	—
Manado	Ma	SEA	22	15	0.90	1.10	−1.94*	−13.49***	—	—
Sangalaki	Sa	SEA	7	3	0.52	1.32	−1.58*	1.60	0.078	0.209
Misool	Mi	SEA	8	5	0.79	1.84	−1.58	−0.16	0.056	0.128
Pulau Seribu	PS	SEA	12	4	0.45	0.29	−1.63*	−2.12**	—	—
Karimunjava	Ka	SEA	20	6	0.52	1.24	−1.31	−0.06	0.067	0.28
Phuket	Ph	EIO	34	13	0.91	1.06	−1.37	−6.90***	—	—
Trang Islands	Tr	EIO	19	6	0.86	0.99	−0.02	−0.80	—	—
Satun Islands	SI	EIO	24	17	0.97	1.57	−1.14	−13.29***	—	—
Padang	Pa	EIO	15	9	0.89	1.26	0.03	−4.12**	—	—
Society Islands	So	CP	12	6	0.68	1.69	−0.82	−0.36	0.053	0.092
French Polynesia	PF	CP	55	55	1.00	3.82	−1.43*	−25.14***	—	—

Abbreviations: *π*, nucleotide diversity; AUS, Australia; CP, Central Pacific; *D*, Tajima's *D*; EIO, Eastern Indian Ocean; Fs, Fu's Fs; *h*, haplotype diversity; HRI, Harpending's Raggedness Index; *n*, number of sequences; *N*_hp_, number of haplotypes; RS, Red Sea; SEA, Seas of Southeastern Asia; SSD, sum of square deviation; WIO, Western Indian Ocean; WP, Western Pacific.

* 0.05 ≥ *p* ≥ 0.01.

** 0.01 > *p* > 0.001.

*** *p* < 0.001.

### 2.2 DNA Extraction, PCR, and Sequencing

Genomic DNA of 35 samples was isolated using the E.Z.N.A. Tissue Genomic DNA Extraction kit (Omega). A fragment of the mitochondrial *COI* gene was employed as the molecular marker, which was amplified utilizing tridacnid‐specific primers: forward 5′‐GGGTGATAATTCGAACAGAA‐3′ and reverse 5′‐TAGTTAAAGCCCCAGCTAAA‐3′ (Kochzius and Nuryanto 2008). PCR reaction was carried out in a total volume of 25 μL containing 10–100 ng of DNA template, 2.5 μL of BIOREADY 10× buffer, 2.0 μL of 10 mM dNTPs, 1.0 μL of each 10 μM primer, 0.2 μL of BIOREADY Taq polymerase, and ddH_2_O to adjust the volume. Amplification of the *COI* gene was performed under the following conditions: initial denaturation at 94°C for 180 s, followed by 35 cycles of 94°C for 60 s, 43°C for 90 s, 72°C for 60 s, and a final extension of 5 min at 72°C. PCR products were purified using the QIAquick PCR purification kit (Qiagen, Germany). Double‐stranded PCR products were sequenced in forward and reverse directions using an ABI 3730 XL automated sequencer (Applied Biosystems).

### 2.3 Sequence Alignment and Genetic Diversity Analysis

To ensure the acquisition of a functional mitochondrial DNA sequence and to minimize the potential sequencing errors, the sequences were translated into amino acids in MEGA 7.0.26 (Kumar et al. 2016) before being subjected to further analysis. Species identity was confirmed by conducting a Blast on GenBank. Other published *COI* sequence data of *T. maxima* across different oceanic regions was downloaded from GenBank (Table S1) (Huelsken et al. 2013; Deboer et al. 2014; Hui, Kraemer, et al. 2016; Hui, Nuryanto, and Kochzius 2016; Keyse et al. 2018; Neo et al. 2018; Fauvelot et al. 2020) and incorporated with the sequences newly generated in this study. All downloaded sequences were further checked to ensure they belonged to *T. maxima* by blasting with *COI* sequences of tridacnid species obtained in our lab, and those sequences showing the highest similarity to other giant clam species rather than *T. maxima* were deleted. Finally, the merged sequences of 610 individuals from 34 locations (Table 1, Figure 1A) were then aligned and trimmed to a uniform length of 417 base pairs using MEGA 7.0.26. Genetic diversity indices, including the number of haplotypes (*N*_hp_), haplotype diversity (*h*) (Nei 1987), and nucleotide diversity (*π*) (Nei and Jin 1989) were calculated using Arlequin v3.0.1 (Excoffier and Lischer 2010).

### 2.4 Genetic Population Structure Analysis

Genetic differentiation among populations was quantified by *COI* sequence divergences and *Φ*_st_ values calculated in Arlequin (Excoffier et al. 1992). *p*‐values were determined from a pseudo‐distribution of *Φ*_st_, which was generated through 10,000 random permutations of the original sequence matrix. Furthermore, a hierarchical analysis of molecular variance (AMOVA) (Excoffier et al. 1992) was conducted in Arlequin to identify spatial groups of samples that exhibited maximum differentiation from one another, indicated by the *Φ*_ct_ value. Further, the best‐fitting model of sequence evolution was identified using ModelFinder (Kalyaanamoorthy et al. 2017), and a maximum‐likelihood (ML) phylogenetic tree with 1000 bootstrap replicates was constructed for all sequences utilizing IQ‐TREE v2.1.2 (Minh et al. 2020). The visualization, manipulation, and annotation of the resulting tree were performed using the iTOL v6.9 online platform (https://itol.embl.de/) (Letunic and Bork 2024).

### 2.5 Gene Flow Analysis

**FIGURE 2:**
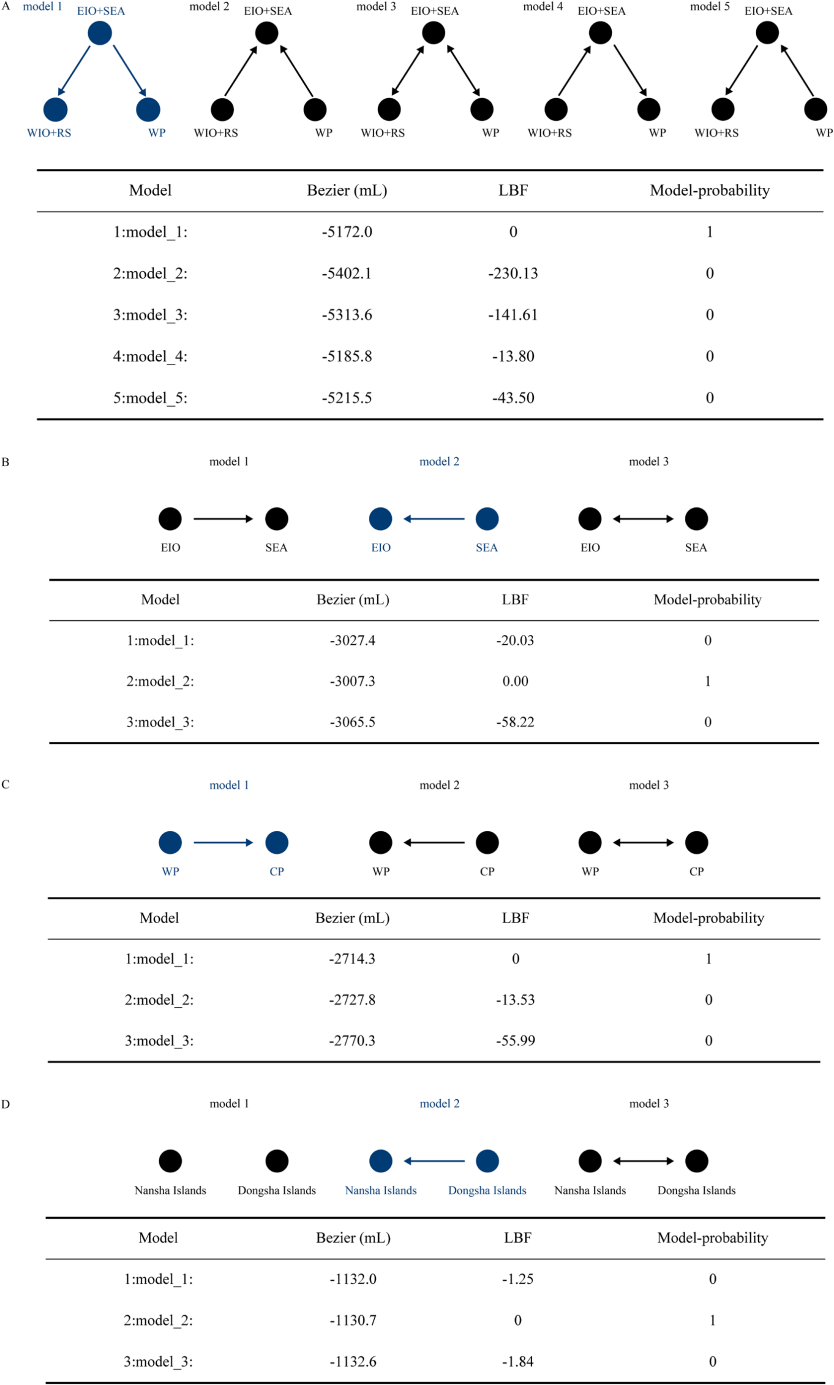
Comparison of various historical gene flow models among different *T. maxima* lineages and populations based on the Bayes factor with MIGRATE‐N software. mL: marginal likelihood. LBF: log Bayes factor. (A) Gene flow model selection among EIO/SEA lineages, WIO/RS linages, and WP linage. (B) Model selection between EIO lineage and SEA lineage. (C) Model selection between WP lineage and CP lineage. (D) Model selection between populations of Nansha Island and Dongsha Island.

MIGRATE‐N v3.6.11 (Beerli and Palczewski 2010) was employed to evaluate various migration scenarios among lineages using distinct migration matrices. Five migration models were set to examine historical gene flow (Figure 2A): (1) migration from the Eastern Indian Ocean (EIO) and the SEA lineages to the Western Indian Ocean (WIO), Red Sea (RS) lineages, and Western Pacific (WP) lineage; (2) migration from the WIO, RS lineages, and WP lineage to the EIO and SEA lineages; (3) symmetrical migration between adjacent sampling locations; (4) migration from the WIO, RS lineages to the EIO, SEA lineages, and then to the WP lineage, proceeding from west to east; and (5) migration from the WP lineage to the EIO, SEA lineages, and then to the WIO, RS lineages, moving from east to west. Additionally, we developed three further migration models to test gene‐flow scenarios between the EIO and SEA (Figure 2B): (1) asymmetrical migration from the EIO to the SEA; (2) asymmetrical migration from the SEA to the EIO; and (3) asymmetrical migration between the EIO and SEA. We also explored alternative scenarios involving gene flow between the WP and CP (Figure 2C), as well as between the Nansha Islands and the Dongsha Islands of the SCS (Figure 2D). The MIGRATE‐N analysis was conducted using default parameters, with adjustments to the run length, heating chain, and transition/transversion ratio for each gene. We utilized a heating scheme of 1.0, 1.5, 3.0, and 1,000,000 to calculate marginal likelihoods for model comparisons. Each model was run twice, with a single long chain of 10,000 steps recorded every 100 generations and discarding the first 100,000 steps as burn‐in. The models were assessed by the Bayes factor (Kass and Raftery 1995).

### 2.6 Historical Demography Analysis

To elucidate the demographic history of *T. maxima* , three complementary methods were applied. Initially, a neutrality test was conducted using Tajima's *D* test (Tajima 1989) and Fu's Fs test (Fu 1997) by Arlequin, each with 10,000 permutations. Subsequently, an analysis of mismatch distributions was performed, utilizing the sum of squared deviations (SSD) and Harpending's Raggedness Index (HRI) (Rogers and Harpending 1992; Rogers 1995) with 10,000 permutations to assess population expansion and the demographic status of the populations. Third, Bayesian skyline plots (BSPs) were implemented using BEAST v2.7.6 (Bouckaert et al. 2014) to reconstruct the demographic dynamics over time. ModelTest‐NG software (Darriba et al. 2020) was utilized to determine the most appropriate substitution model for the *COI* sequence data of the RS, WIO, EIO, SEA, WP, and CP lineages, which were then analyzed separately with the HKY, HKY+G8, HKY+G8, HKY+G8, HKY+I, and HKY+G8 models, respectively, in conjunction with constant Bayesian skyline tree priors. A strict molecular clock model with a rate of 1.2% per million years (Ma) (Marko 2002) was used to convert substitution rates per site into calendar years. Six independent Markov chain Monte Carlo (MCMC) analyses were conducted in BEAST, each with a chain length of 30,000,000 steps and a sampling frequency of every 3000 generations, discarding the first 10% of the chain as burn‐in. The effective sample size for key parameters sampled in the MCMC was checked to ensure it was greater than 200, and the results were visualized using Tracer v1.7.2 (Rambaut et al. 2018).

### 2.7 Ecological Niche Analysis

Present and future potential distribution of *T. maxima* was predicted using SDM. The distribution records for the *T. maxima* were obtained by combining our sampling data, searching published literature, and online databases OBIS (Ocean Biodiversity Information System, https://obis.org/) and GBIF (Global Biodiversity Information Facility, https://doi.org/10.15468/dl.w5huzy). It was found that the distribution range spans the tropical and temperate nearshore from 40° S to 40° N. To reduce the effects of sampling bias on model fitting, we kept only one distribution record per 0.05° grid cell, matching the resolution of environmental parameters. After data filtering, 177 distribution records for *T. maxima* were retained, with 75% of them being used to construct the maximum entropy model, and the remaining 25% for testing. We retrieved 21 environmental variables from Bio‐ORACLE v3.0 (https://bio‐oracle.org/) and checked collinearity among predictors via the pairwise Pearson's correlation coefficients (*r*) (Figure S1). Only one among highly correlated predictors with a threshold for collinearity at |*r*| > 0.7 (Dormann et al. 2013) was kept on the basis of present‐day and future data availability, biological importance, and their contributions in pre‐modeling for SDM analysis. Finally, six predictors were retained for subsequent analysis: depth, annual mean benthic temperature, annual mean benthic dissolved oxygen concentration, annual mean benthic salinity, and annual mean benthic current velocity. To characterize the future habitat suitability, we considered future marine climate under two Shared Socioeconomic Pathway (SSP) scenarios, SSP 1.19 and SSP 5.85, in the 2090s (average of 2090–2100). The future environmental variables were also downloaded from Bio‐ORACLE.

We modeled habitat suitability for *T. maxima* using the six abiotic predictors (hereafter abiotic‐only model) via MaxEnt version 3.4.1 (Phillips and Dudík 2008) and evaluated the model accuracy by the receiver operating characteristic curve (ROC) and area under the curve (AUC). The ROC value of the AUC ranged from 0 to 1 and had a positive correlation with the model accuracy (Hanley and Mcneil 1983). The Jenks Natural Breaks method (Jenks 1967) implemented in Geographic Information Systems ArcGIS v. 10.8 was utilized to classify the area into four distinct suitable levels: non‐suitable zone, low‐suitable zone, medium‐suitable zone, and high‐suitable zone. The core principle of this method was to maximize the variance between groups while minimizing the variance within groups. Additionally, it was reported that when the temperature was higher than 32°C, the giant clams exhibited metabolic disorder and impaired physiological functions, while the heightened mortality of zooxanthellae resulted in the loss of photosynthetic products, ultimately leading to the death of the clams (Dubousquet et al. 2016). Therefore, we set an average temperature upper limit of 32°C for the hottest month. It was supposed that if it was higher than the limit, the area could not be suitable for the survival of the clams. The results of the MaxEnt model were finally visualized by ArcGIS v. 10.8.

## 3 Results

### 3.1 Genetic Diversity

A total of 35 *COI* sequences of *T. maxima* from the Nansha Islands of the SCS were newly obtained and deposited in GenBank with accession numbers PQ821125–PQ821159. The integration of our data with previously published sequence data from GenBank yielded a total of 610 *COI* sequences for *T. maxima* , which encompassed 360 unique haplotypes and 171 polymorphic sites. The sample range extended across various regions, including China's Nansha and Dongsha Islands, as well as the RS, WIO, EIO, SEA, Solomon Islands, and the Society Islands in the CP, covering 34 populations (Table 1, Figure 1A). Each population exhibited a high level of genetic diversity, with overall *h* = 0.84 and *π* = 1.86%. The number of haplotypes ranged from three (Reunion Island and Sangalaki population) to 55 (French Polynesia). The *h* varied from 0.45 (Pulau Seribu) to a maximum of 1.00 (South of Philippines, North of Philippines, Solomon Islands, and French Polynesia). Nucleotide diversity ranged from 0.29% in Pulau Seribu to 7.12% in Biak. Overall, the populations from Pulau Seribu, Sangalaki, and Karimunjava displayed lower genetic diversity (*h* < 0.55, *π* < 1.35%) compared to the other populations. In the SCS, population genetic diversity indices were as follows: the Dongsha Islands (*h* = 0.85, *π* = 2.28%), Meiji Reef in the Nansha Islands (*h* = 0.76, *π* = 0.88%), and Yongshu Reef in the Nansha Islands (*h* = 0.78, *π* = 0.84%). In comparison, the genetic diversity of populations from the Nansha Islands was lower than that of the Dongsha Islands.

### 3.2 Genetic Population Structure

Highly significant genetic population structure in *T. maxima* was confirmed by AMOVA and phylogenetic analysis. Hierarchical AMOVA identified the highest fixation index (*Φ*_st_ = 0.856, *Φ*_ct_ = 0.836, *p* = 0.000) with 83.55% variation among groups when sample sites were assigned to the following regions from west to east: (1) RS, (2) WIO (Kenya, Juan de Nova, Tulear, Reunion Island), (3) EIO (Phuket, Trang Islands, Satun Islands, Padang, Pulau Seribu, Karimunjava), (4) SEA (Yongshu Reef, Meiji Reef, Dongsha Islands, North of Philippines, South of Philippines, Manado, Sangalaki, Togian Islands, Luwuk, Sembilan Islands, Spermonde, Bira, Komodo, and Kupang), (5) WP (Biak, Solomon Islands, Torres Strait, Lizard Island, Lihou Reef, Heron Island), and (6) CP (Society Islands, French Polynesia) (Figure 1A). The relationship among the 34 populations was further elucidated by the phylogenetic tree (Figure 1B). The 610 individuals were clustered into six groups (Figure 1C). Notably, the SEA group was predominantly located along the Indonesian Throughflow (ITF), and individuals from the Nansha and Dongsha Islands in the SCS were intermixed and formed part of the SEA cluster.

### 3.3 Gene Flow

We simulated scenarios for multiple models of population migration using coalescent approaches as implemented in MIGRATE‐N. The populations of *T. maxima* were first divided into three major groups according to the geographic distribution: (1) the WIO and the RS, (2) the EIO and the SEA, and (3) the WP, to investigate extensive gene flow. Model selection identified model 1 as the best‐fitting based on the largest Bezier value (Figure 2), which suggested that migration was more inclined to originate from the EIO and the SEA lineages and flow to the WIO and the RS lineages, as well as the WP lineage. Further analysis of gene flow between the EIO and the SEA revealed that model 2 was the optimal model, suggesting migration direction from the SEA to the EIO. Moreover, gene flow was determined from the WP to the CP in the Pacific populations, while the model of migration direction from the Dongsha Islands to the Nansha Islands was identified as the best fit in the SCS.

### 3.4 Historical Demography

**FIGURE 3:**
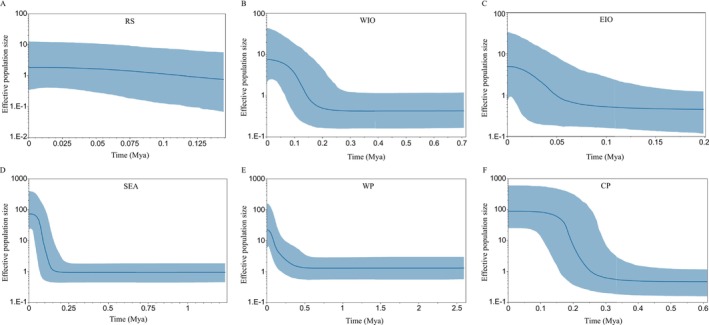
Bayesian skyline plot of *T. maxima* from different regions. The solid blue line shows the median estimates, and the blue area represents the 95% of the probability of the posterior distribution (95% HPD). MyA, million years ago. A‐E: Bayesian skyline plot for RS lineage (A), WIO lineage (B), EIO lineage (C), SEA lineage (D), WP lineage (E), and CP lineage (F).

The majority of Tajima's *D* tests and Fu's Fs tests for each population resulted in the rejection of the null hypothesis of neutral evolution for the *COI* marker, with most populations showing significant negative values for both tests, indicative of a significant departure from mutation‐drift equilibrium, particularly pronounced in Fu's Fs (Table 1). The mismatch distribution analysis and Rogers' test of sudden population expansion showed that populations experienced expansion (Rogers 1995) (Table 1). Further, BSP confirmed that significant demographic expansion events occurred in *T. maxima* during the last 500,000 to 75,000 years in the WIO, EIO, SEA, WP, and CP lineages (Figure 3). However, the effective population size of *T. maxima* in the RS lineage remained relatively stable over the past 150,000 years.

### 3.5 Ecological Niche Distribution

**FIGURE 4:**
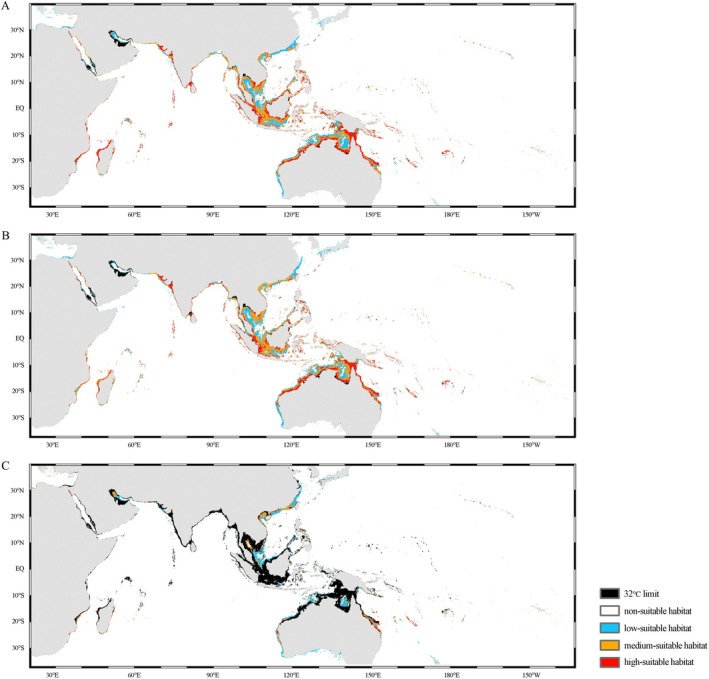
Suitable distribution areas of *T. maxima* . (A) Distribution under current situation. (B) Distribution with SSP 1.19 in 2090s. (C) Distribution with SSP 5.85 in 2090s.

The AUC value of the ecological niche model for *T. maxima* generated by the MaxEnt was 0.990, indicating that the model had a high level of predictive accuracy. Among all the environmental factors incorporated into the modeling, depth, annual mean benthic temperature, and annual mean benthic dissolved oxygen concentration showed the strongest influence on the occurrence of *T. maxima* (Table 2). The habitat suitability for *T. maxima* was categorized into four tiers by the Jenks Natural Breaks method: 0–0.0926 for non‐suitable habitat, 0.0926–0.2806 for low‐suitable habitat, 0.2806–0.4658 for medium‐suitable habitat, and 0.4658–1 for high‐suitable habitat (Derolph et al. 2015). The SDM results showed that under the current situation, the small giant clam was extensively distributed in shallow coral reef areas of the tropical region within the WP and Indian Ocean, encompassing the RS, the SCS, Kepulauan Sunda Besar, the Ryukyu Islands, and North Australia (Figure 4A), which was largely consistent with actual observations. A notable reduction in high‐suitable habitats for *T. maxima* was detected from nearshore to offshore areas in the coastal zones. The current high‐suitable habitat area was 2,156,336.9 km^2^ under the present scenarios, which was projected to decrease to 2,134,588.9 km^2^ under the scenarios SSP 1.19, 2090s, and to 222,529.8 km^2^ under the scenarios SSP 5.85, 2090s (Table 3), reflecting a sharp decline as environmental pollution levels increased (Figure 4B,C). The prediction showed that the distribution of the giant clams would slightly move to a higher latitude under both scenarios (Figure 4B,C).

**TABLE 2:** Environmental variable importance.

Variable	Contribution (%)	Permutation importance (%)
depth	63.1	2.4
tem	32.6	85.6
do	2.3	7.6
sal	1.9	3.7
cur	0	0.7

Abbreviations: cur, annual mean benthic current velocity; do, annual mean benthic dissolved oxygen concentration; sal, annual mean benthic salinity; tem, annual mean benthic temperature.

**TABLE 3:** Distribution area of *T. maxima* under three scenarios.

Scenarios	Suitability level	Area (km^2^)	Net change
Present	Non‐suitable habitat	235,642,853.6	
	Low‐suitable habitat	2,482,804.9	
	Medium‐suitable habitat	2,420,844.7	
	High‐suitable habitat	2,156,336.9	
SSP 1.19	Non‐suitable habitat	235,567,974.7	−0.03%
	Low‐suitable habitat	2,454,427.1	−1.14%
	Medium‐suitable habitat	2,404,115.5	−0.69%
	High‐suitable habitat	2,134,588.9	−1.022%
SSP 5.85	Non‐suitable habitat	235,068,018.3	−0.24%
	Low‐suitable habitat	1,892,200.8	−23.78%
	Medium‐suitable habitat	731,160.6	−69.79%
	High‐suitable habitat	222,529.8	−89.68%

## 4 Discussion

### 4.1 Maintenance of the Population Genetic Pattern: The Current

This research compiles *COI* data of *T. maxima* from different oceanic regions available on GenBank and supplements it with samples from the Nansha Islands of the SCS. The expanded dataset enables a large‐scale biogeographical analysis of *T. maxima* across an extended geographical range. A distinct and well‐defined genetic population structure of *T. maxima* throughout the Indo‐Pacific region is disclosed. However, there are limitations to conducting population genetic analysis solely with mitochondrial *COI* or a few nuclear markers, which cannot capture genome‐wide diversity and fully reveal the genetic structure. Population genomics studies based on genome re‐sequencing are anticipated to yield deeper insights into the microevolution of *T. maxima* . However, to gain a comprehensive understanding of the genetic structure of *T. maxima* across its entire distribution range, *COI* stands out as the most suitable marker at present, given the extensive published *COI* data from various regions.

In this study, six lineages of *T. maxima* are identified from west to east, exhibiting pronounced genetic differentiation. Similar patterns of genetic differentiation have also been detected in other tridacnid species (Huelsken et al. 2013; Deboer et al. 2014; Hui, Kraemer, et al. 2016; Hui, Nuryanto, and Kochzius 2016) and invertebrates, such as *Linckia laevigata* (Otwoma and Kochzius 2016) and *Acropora millepora* (Van Der Ven et al. 2021). It is found that the current population genetic patterns are closely associated with geographical barriers, geographical distance, and ocean currents. For instance, the RS and the Indian Ocean are only connected by the Bab‐el‐Mandeb Strait, which might restrict population connectivity (Dibattista et al. 2016). Genetic differentiation of the giant clam between the western and EIO is mostly due to the vast oceanic distances between them, as well as between WP and CP. The genetic differentiation between SEA populations and those in the EIO may be attributed to the lowering of sea levels during glacial periods and the exposure of the Sunda Shelf, as well as the barrier effect of Sumatra and Java (Voris 2020). In contrast, the strong genetic connectivity observed in SEA is closely associated with the ITF. Likewise, the ocean currents in the SCS also connect the populations in the SCS with those in the ocean currents in the IMA (Figure 1C). Meanwhile, the North Equatorial Counter Current contributes to the genetic separation between populations in the WP and those in SEA (Wyrtki 1961, Gordon and Fine 1996).

### 4.2 History Formation Process of the Population Genetic Pattern: The Past

Nevertheless, the process through which the current distribution pattern of *T. maxima* has been formed remains elusive. By modeling historical gene flow, our findings suggest that gene flow has dispersed both eastward and westward along the SEA, exhibiting a general migratory trend that radiates outward from the CT to its periphery. This provides compelling evidence in support of the hypothesis that the CT serves as a central origin for species diversity. However, genetic flow patterns across species in Indo‐Pacific regions exhibit considerable variability. For instance, some species, such as the fish *Acanthurus nigricans* , provide evidence supporting the “Centre of diversity origin” hypothesis, while others, like the echinoderms *Holothuria atra* and *Acanthaster planci* , do not (Matias and Riginos 2018). These findings indicate that the evolutionary and biogeographic patterns of species are influenced by a complex interplay of various factors, not only ecological and life history traits but also species‐specific historical events, geographic distributions, and population dynamics. Specifically, in examining the gene‐flow direction of giant clams in the SCS, the optimal model indicates a migratory path from the Dongsha Islands to the Nansha Islands. It is postulated to be a consequence of the ITF and the SCS monsoon (Morton and Blackmore 2001; Hu et al. 2015), which act to transport planktonic larvae in a north‐to‐south direction, ultimately leading them towards the Nansha Islands (Figure 1C).

Moreover, spatial expansion of *T. maxima* is detected in most populations by Tajima's *D* and Fu's Fs tests, which is further substantiated by the demographic history deduced from coalescent simulation analysis and BSPs. The estimated timings of expansion exhibited slight variations among different lineages of *T. maxima* , ranging from 0.07 to 0.50 MYA, which fall within the range of the late Pleistocene with nine glacial cycles recorded in the last 800,000 years (Hobart et al. 2023). Correlations between population expansion and Pleistocene sea‐level changes have been reported in another giant clam species, *Tridacna noae*, as well as many other Indo‐Pacific reef‐associated invertebrates and fish (Ludt et al. 2012; Delrieu‐Trottin et al. 2017; Fauvelot et al. 2019; Wang, Zhang, et al. 2022; Wang, Wu, et al. 2022; Gu et al. 2022). The significant phylogeographic structure in conjunction with the evidence of spatial expansion indicates there might be cryptic species and an allopatric divergence. It is presumed that the sea‐level fall in the late Pleistocene glaciation had a profound impact on the shallow‐water reef habitats that are conducive to the survival of giant clams (Lambeck and Chappell 2001), and viable populations of numerous Indo‐Pacific reef‐associated organisms managed to endure in isolated refuges (Pellissier et al. 2014). Upon the subsequent rise in sea levels, the surviving populations, which had already developed differentiated lineages, re‐expanded to attain their current geographical distributions. However, the RS lineage demonstrated a sluggish and marginal increase in effective population size without a pronounced population expansion, as inferred from the BSP. In a situation like that of RS, it is recommended that further analysis based on multilocus data may enhance the ability and precision of recovering population size dynamics that may not be fully characterized by using a single gene marker (Heled and Drummond 2008).

### 4.3 Distribution Change Under Climate Change and Indication for Conservation: The Future

Climate change, particularly the rise in temperature, exerts a significant impact on the physiological processes of giant clams and results in widespread mortality (Dubousquet et al. 2016; Zhou et al. 2019). The mass bleaching events of giant clams in the Philippines in 1998 (Gomez and Mingoa‐Licuanan 1998) and in the Gulf of Thailand in 2010 (Junchompoo et al. 2013) highlight the adverse effects of increasing seawater temperatures. In our study, model analysis reveals that the annual mean benthic temperature is most closely associated with the distribution of giant clams, an environmental factor recognized as crucial in ecological studies (Blidberg et al. 2000; Buck et al. 2002; Zhou et al. 2019; Brahmi et al. 2021). Currently, the high suitability areas of *T. maxima* are primarily found in the coral reefs of tropical regions in the Indo‐Pacific oceans. The SDM has demonstrated good predictive performance in areas where *T. maxima* has been previously recorded, with results largely consistent with actual observations. However, there is a discrepancy between the predicted and observed distributions. For instance, the SDM has identified the Caribbean Sea and the Gulf of Mexico with coral reefs as potential suitable habitat for *T. maxima* , but no records of *T. maxima* have been documented in the Atlantic Ocean to date. Considering that the planktonic period of *T. maxima* lasts for only about 10 days before settling on a substrate as a juvenile (Soo and Todd 2014), the absence of *T. maxima* in the Atlantic regions might be attributed to geographical isolation. Thus, we cannot solely rely on the results of the SDM to determine the distribution of the species, and it is also necessary to consider the ecological habit and biological characteristics in order to reasonably interpret the SDM results.

Critically, it is predicted that as climate change leads to global warming, the high‐suitable habitat is projected to sharply decline by 2090 due to escalating environmental pollution, a trend also observed in corals (Sully et al. 2022). Of course, we also need to consider the adaptive evolution of organisms, and the reduction in the distribution range might be overestimated. Moreover, it is found that global warming has prompted many terrestrial and marine species to exploit the reduced temperature gradient from the equator to the poles, expanding their ranges poleward (Pecl et al. 2017). A subtle trend of migration towards higher latitudes is also noted in the distribution of *T. maxima* . Under the most severe climate change scenario, the southernmost boundary of the *T. maxima* suitable habitat would possibly shift from around 25° S to around 30° S. The SDM predictions indicate that the nearshore areas of southern China mainland, Gulf of Suez, and eastern Australia with coral reefs (Lyons et al. 2024) might still be high‐suitable habitat for small giant clams under the SSP 5.85 scenario by 2090. However, current conditions reveal that the nearshore areas of southern mainland China, like Hainan, and the Gulf of Suez are under severe pressure, such as habitat degradation and a range of intensive human activities (Hughes et al. 2013; Hasan 2018; Chen et al. 2021). On the other hand, the Great Barrier Reef in Australia has been reported as one of the regions most severely affected by marine heatwaves, with its coral communities experiencing multiple large‐scale bleaching events (Pratchett et al. 2021). Additionally, in the SDM analysis, only a limited number of environmental variables, like water temperature, are used; other ecological factors such as suitable substrate (coral reefs) and water quality (nutrients, heavy metal etc.), should also be considered in order to more exactly determine the distribution of giant clams. Nonetheless, the prediction does reflect the shrinking distribution range of the giant clam with global warming.

Undoubtedly, the future of giant clams is precarious. The global orchestration of conservation initiatives for different genetic lineages and suitable habitat of *T. maxima* is vital by incorporating these areas into existing global conservation plans (Beyer et al. 2018; Gil‐Agudelo et al. 2020). Moreover, should the Paris Agreement's emission reduction targets not be met, many coral reefs and their associated organisms are at risk of disappearing in the latter half of this century (Donner et al. 2005; Frieler et al. 2013; Van Hooidonk et al. 2016). Therefore, addressing climate change, including global warming, is the priority for coral reef ecosystem protection (Hughes et al. 2003).

## 5 Conclusions

In summary, a pronounced genetic population structure of *T. maxima* across the Indo‐Pacific region has been identified, shedding light on its connections with geological history, fluctuations in sea levels, and oceanographic variables. By inferring source‐sink relationships among populations through the gene‐flow patterns, the research bolsters the theory of a center of origin, accounting for the elevated biodiversity of the CT. The high‐suitable habitat is predicted to sharply decline and move towards higher latitudes under future climate change, with global warming as a principal component posing a significant threat to the distribution of *T. maxima* . It is proposed to conserve diverse genetic resources and suitable habitat of *T. maxima* , and improve the global climate through coordinated environmental protection efforts.

## Author Contributions

**Haojun Chi:** formal analysis (lead), investigation (lead), writing – original draft (equal). **Zhongli Sha:** conceptualization (equal), funding acquisition (equal), writing – review and editing (equal). **Lin He:** formal analysis (equal), resources (equal), writing – review and editing (equal). **Min Hui:** conceptualization (equal), funding acquisition (equal), resources (equal), supervision (lead), writing – original draft (equal), writing – review and editing (equal).

## Ethics Statement

This research was conducted under the project of “Research on the Principles of Biodiversity Conservation in the Coral Reef Ecosystem of the South China Sea” (2021YFF0502801) approved by the Ministry of Science and Technology of China. By SCUBA diving, very small mantle tissues of giant clams were cut using a biopsy method to ensure the survival of the clams.

## Consent

The authors have nothing to report.

## Conflicts of Interest

The authors declare no conflicts of interest.

## Supporting information


**Data S1:** ece371965‐sup‐0001‐FigureS1.pdf.

## Data Availability

The COI sequences obtained in the present study are available on GenBank: PQ821125–PQ821159.
