# Supplementary material for: Genetic Population Structure and Distribution of the Small Giant Clam Tridacna maxima in Indo‐Pacific Coral Reefs: History Dynamics, Present Status and Future Trends
Source: Ecol Evol. 2025 Aug 8;15(8):e71965. doi: 10.1002/ece3.71965 (PMC12334851; doi:10.1002/ece3.71965)
Supplement: Supplementary file 1 — Data S1: ece371965‐sup‐0001‐FigureS1.pdf. [file ECE3-15-e71965-s001.pdf]

Supplementary material

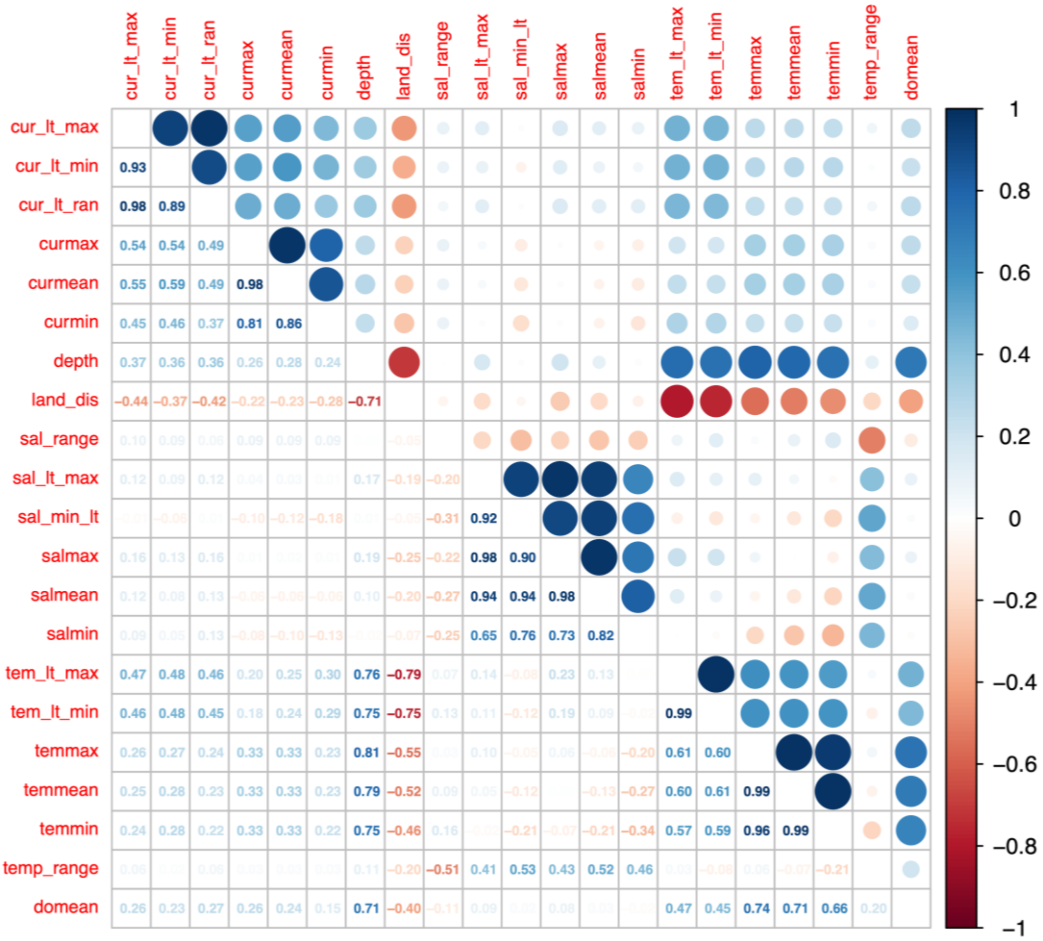

Supplementary Figure 1 Results of collinearity analysis of 21 predictors. cur_lt_max, annual average of the maximum benthic current velocity; cur_lt_min, annual average of the minimum benthic current velocity; cur_lt_ran, annual range of long-term benthic current velocity; curmax, annual maximum benthic current velocity; curmean, annual mean benthic current velocity; curmin, annual minimum benthic current velocity; depth, depth; land_dis, distance to land; sal_range, annual range of benthic salinity; sal_lt_max, annual average of the maximum benthic salinity; sal_min_lt, annual average of the minimum benthic salinity; salmax, annual maximum benthic salinity; salmean, annual mean benthic salinity; salmin, annual minimum benthic salinity; tem_lt_max, annual average of the maximum benthic temperature; tem_lt_min, annual average of the minimum benthic temperature; temmax, annual maximum benthic temperature; temmean, annual mean benthic temperature; temmin, annual minimum

benthic temperature; temp_range, annual range of benthic temperature; domean, annual mean benthic dissolved oxygen concentration.

Supplementary Table 1. Genbank accession numbers for all sequences used in this work

Individual label	Sampling Location	Accession no.	Reference
PF55	French Polynesia	MF167520.1	Dubousquet et al., 2014
PF54	French Polynesia	MF167519.1	Dubousquet et al., 2014
PF53	French Polynesia	MF167518.1	Dubousquet et al., 2014
PF52	French Polynesia	MF167517.1	Dubousquet et al., 2014
PF51	French Polynesia	MF167516.1	Dubousquet et al., 2014
PF50	French Polynesia	MF167515.1	Dubousquet et al., 2014
PF49	French Polynesia	MF167514.1	Dubousquet et al., 2014
PF48	French Polynesia	MF167513.1	Dubousquet et al., 2014
PF47	French Polynesia	MF167512.1	Dubousquet et al., 2014
PF46	French Polynesia	MF167511.1	Dubousquet et al., 2014
PF45	French Polynesia	MF167510.1	Dubousquet et al., 2014
PF44	French Polynesia	MF167509.1	Dubousquet et al., 2014
PF43	French Polynesia	MF167508.1	Dubousquet et al., 2014
PF42	French Polynesia	MF167507.1	Dubousquet et al., 2014
PF41	French Polynesia	MF167506.1	Dubousquet et al., 2014
PF40	French Polynesia	MF167505.1	Dubousquet et al., 2014
PF39	French Polynesia	MF167504.1	Dubousquet et al., 2014
PF38	French Polynesia	MF167503.1	Dubousquet et al., 2014
PF37	French Polynesia	MF167502.1	Dubousquet et al., 2014
PF36	French Polynesia	MF167501.1	Dubousquet et al., 2014
PF35	French Polynesia	MF167500.1	Dubousquet et al., 2014
PF34	French Polynesia	MF167499.1	Dubousquet et al., 2014
PF33	French Polynesia	MF167498.1	Dubousquet et al., 2014
PF32	French Polynesia	MF167497.1	Dubousquet et al., 2014
PF31	French Polynesia	MF167496.1	Dubousquet et al., 2014
PF30	French Polynesia	MF167495.1	Dubousquet et al., 2014
PF29	French Polynesia	MF167494.1	Dubousquet et al., 2014
PF28	French Polynesia	MF167493.1	Dubousquet et al., 2014

PF27	French Polynesia	MF167492.1	Dubousquet et al., 2014
PF26	French Polynesia	MF167491.1	Dubousquet et al., 2014
PF25	French Polynesia	MF167490.1	Dubousquet et al., 2014
PF24	French Polynesia	MF167489.1	Dubousquet et al., 2014
PF23	French Polynesia	MF167488.1	Dubousquet et al., 2014
PF22	French Polynesia	MF167487.1	Dubousquet et al., 2014
PF21	French Polynesia	MF167486.1	Dubousquet et al., 2014
PF20	French Polynesia	MF167485.1	Dubousquet et al., 2014
PF19	French Polynesia	MF167484.1	Dubousquet et al., 2014
PF18	French Polynesia	MF167483.1	Dubousquet et al., 2014
PF17	French Polynesia	MF167482.1	Dubousquet et al., 2014
PF16	French Polynesia	MF167481.1	Dubousquet et al., 2014
PF15	French Polynesia	MF167480.1	Dubousquet et al., 2014
PF14	French Polynesia	MF167479.1	Dubousquet et al., 2014
PF13	French Polynesia	MF167478.1	Dubousquet et al., 2014
PF12	French Polynesia	MF167477.1	Dubousquet et al., 2014
PF11	French Polynesia	MF167476.1	Dubousquet et al., 2014
PF10	French Polynesia	MF167475.1	Dubousquet et al., 2014
PF9	French Polynesia	MF167474.1	Dubousquet et al., 2014
PF8	French Polynesia	MF167473.1	Dubousquet et al., 2014
PF7	French Polynesia	MF167472.1	Dubousquet et al., 2014
PF6	French Polynesia	MF167471.1	Dubousquet et al., 2014
PF5	French Polynesia	MF167470.1	Dubousquet et al., 2014
PF4	French Polynesia	MF167469.1	Dubousquet et al., 2014
PF3	French Polynesia	MF167468.1	Dubousquet et al., 2014
PF2	French Polynesia	MF167467.1	Dubousquet et al., 2014
PF1	French Polynesia	MF167466.1	Dubousquet et al., 2014
TmDQ	Dongsha Islands	MG195278.1	Neo et al., 2018
Tm21	Dongsha Islands	MG195277.1	Neo et al., 2018
Tm20	Dongsha Islands	MG195276.1	Neo et al., 2018
Tm19	Dongsha Islands	MG195275.1	Neo et al., 2018
Tm18	Dongsha Islands	MG195274.1	Neo et al., 2018
Tm17	Dongsha Islands	MG195273.1	Neo et al., 2018
Tm16	Dongsha Islands	MG195272.1	Neo et al., 2018
Tm15	Dongsha Islands	MG195271.1	Neo et al., 2018
Tm14	Dongsha Islands	MG195270.1	Neo et al., 2018
Tm8	Dongsha Islands	MG195269.1	Neo et al., 2018
Tm6	Dongsha Islands	MG195268.1	Neo et al., 2018
NML_DS27	Dongsha Islands	MG195223.1	Neo et al., 2018

NML_DS24	Dongsha Islands	MG195222.1	Neo et al., 2018
NML_DS22	Dongsha Islands	MG195221.1	Neo et al., 2018
NML_DS34	Dongsha Islands	MG195220.1	Neo et al., 2018
NML_DS52	Dongsha Islands	MG195219.1	Neo et al., 2018
NML_DS12	Dongsha Islands	MG195218.1	Neo et al., 2018
NML_DS10	Dongsha Islands	MG195217.1	Neo et al., 2018
NML_DS29	Dongsha Islands	MG195216.1	Neo et al., 2018
NML_DS62	Dongsha Islands	MG195215.1	Neo et al., 2018
NML_DS51	Dongsha Islands	MG195214.1	Neo et al., 2018
NML_DS26	Dongsha Islands	MG195213.1	Neo et al., 2018
NML_DS21	Dongsha Islands	MG195212.1	Neo et al., 2018
NML_DS07	Dongsha Islands	MG195211.1	Neo et al., 2018
NML_DS05	Dongsha Islands	MG195210.1	Neo et al., 2018
NML_DS48	Dongsha Islands	MG195209.1	Neo et al., 2018
NML_DS43	Dongsha Islands	MG195208.1	Neo et al., 2018
NML_DS33	Dongsha Islands	MG195207.1	Neo et al., 2018
NML_DS32	Dongsha Islands	MG195206.1	Neo et al., 2018
NML_DS31	Dongsha Islands	MG195205.1	Neo et al., 2018
NML_DS23	Dongsha Islands	MG195204.1	Neo et al., 2018
NML_DS08	Dongsha Islands	MG195203.1	Neo et al., 2018
NML_DS49	Dongsha Islands	MG195202.1	Neo et al., 2018
NML_DS09	Dongsha Islands	MG195201.1	Neo et al., 2018
NML_DS30	Dongsha Islands	MG195200.1	Neo et al., 2018
NML_DS35	Dongsha Islands	MG195199.1	Neo et al., 2018
NML_DS36	Dongsha Islands	MG195198.1	Neo et al., 2018
NML_DS44	Dongsha Islands	MG195197.1	Neo et al., 2018
NML_DS41	Dongsha Islands	MG195196.1	Neo et al., 2018
TOR_2019	Torres Strait	MG385480.1	Keyse et al., 2018
TOR_2018	Torres Strait	MG385479.1	Keyse et al., 2018
TOR_2017	Torres Strait	MG385478.1	Keyse et al., 2018
TOR_2016	Torres Strait	MG385477.1	Keyse et al., 2018
TOR_2015	Torres Strait	MG385476.1	Keyse et al., 2018
TOR_2013	Torres Strait	MG385475.1	Keyse et al., 2018
TOR_2011	Torres Strait	MG385474.1	Keyse et al., 2018
TOR_2006	Torres Strait	MG385473.1	Keyse et al., 2018
TOR_1998	Torres Strait	MG385472.1	Keyse et al., 2018
TOR_1997	Torres Strait	MG385471.1	Keyse et al., 2018
TOR_1996	Torres Strait	MG385470.1	Keyse et al., 2018
TOR_1995	Torres Strait	MG385469.1	Keyse et al., 2018

TOR_1993	Torres Strait	MG385468.1	Keyse et al., 2018
TOR_1964	Torres Strait	MG385467.1	Keyse et al., 2018
TOR_1949	Torres Strait	MG385466.1	Keyse et al., 2018
TOR_1932	Torres Strait	MG385465.1	Keyse et al., 2018
TOR_1931	Torres Strait	MG385464.1	Keyse et al., 2018
TOR_1923	Torres Strait	MG385463.1	Keyse et al., 2018
TOR_1922	Torres Strait	MG385462.1	Keyse et al., 2018
TOR_1921	Torres Strait	MG385461.1	Keyse et al., 2018
TOR_1896	Torres Strait	MG385460.1	Keyse et al., 2018
LIZ_666	Lizard Island	MG385412.1	Keyse et al., 2018
LIZ_665	Lizard Island	MG385411.1	Keyse et al., 2018
LIZ_664	Lizard Island	MG385410.1	Keyse et al., 2018
LIZ_662	Lizard Island	MG385409.1	Keyse et al., 2018
LIZ_661	Lizard Island	MG385408.1	Keyse et al., 2018
LIZ_658	Lizard Island	MG385407.1	Keyse et al., 2018
LIZ_653	Lizard Island	MG385406.1	Keyse et al., 2018
LIZ_652	Lizard Island	MG385405.1	Keyse et al., 2018
LIZ_651	Lizard Island	MG385404.1	Keyse et al., 2018
LIZ_648	Lizard Island	MG385403.1	Keyse et al., 2018
LIZ_2828	Lizard Island	MG385402.1	Keyse et al., 2018
LIZ_2827	Lizard Island	MG385401.1	Keyse et al., 2018
LIZ_2826	Lizard Island	MG385400.1	Keyse et al., 2018
LIZ_2795	Lizard Island	MG385399.1	Keyse et al., 2018
LIZ_2794	Lizard Island	MG385398.1	Keyse et al., 2018
LIZ_2792	Lizard Island	MG385397.1	Keyse et al., 2018
LIZ_2790	Lizard Island	MG385396.1	Keyse et al., 2018
LIZ_2789	Lizard Island	MG385395.1	Keyse et al., 2018
LIH_376	Lihou Reef	MG385394.1	Keyse et al., 2018
LIH_373	Lihou Reef	MG385393.1	Keyse et al., 2018
LIH_356	Lihou Reef	MG385392.1	Keyse et al., 2018
LIH_310	Lihou Reef	MG385391.1	Keyse et al., 2018
LIH_307	Lihou Reef	MG385390.1	Keyse et al., 2018
LIH_306	Lihou Reef	MG385389.1	Keyse et al., 2018
LIH_304	Lihou Reef	MG385388.1	Keyse et al., 2018
LIH_302	Lihou Reef	MG385387.1	Keyse et al., 2018
LIH_300	Lihou Reef	MG385386.1	Keyse et al., 2018
HER_3139	Heron Island	MG385373.1	Keyse et al., 2018
HER_3138	Heron Island	MG385372.1	Keyse et al., 2018
HER_3137	Heron Island	MG385371.1	Keyse et al., 2018

HER_3136	Heron Island	MG385370.1	Keyse et al., 2018
HER_3135	Heron Island	MG385369.1	Keyse et al., 2018
HER_3134	Heron Island	MG385368.1	Keyse et al., 2018
HER_3133	Heron Island	MG385367.1	Keyse et al., 2018
HER_3132	Heron Island	MG385366.1	Keyse et al., 2018
HER_3131	Heron Island	MG385365.1	Keyse et al., 2018
HER_3130	Heron Island	MG385364.1	Keyse et al., 2018
HER_3129	Heron Island	MG385363.1	Keyse et al., 2018
HER_3128	Heron Island	MG385362.1	Keyse et al., 2018
HER_3127	Heron Island	MG385361.1	Keyse et al., 2018
HER_3126	Heron Island	MG385360.1	Keyse et al., 2018
HER_3125	Heron Island	MG385359.1	Keyse et al., 2018
HER_3121	Heron Island	MG385358.1	Keyse et al., 2018
HER_3120	Heron Island	MG385357.1	Keyse et al., 2018
HER_3119	Heron Island	MG385356.1	Keyse et al., 2018
HER_3118	Heron Island	MG385355.1	Keyse et al., 2018
HER_3117	Heron Island	MG385354.1	Keyse et al., 2018
HER_3116	Heron Island	MG385353.1	Keyse et al., 2018
HER_3115	Heron Island	MG385352.1	Keyse et al., 2018
HER_3114	Heron Island	MG385351.1	Keyse et al., 2018
HER_3113	Heron Island	MG385350.1	Keyse et al., 2018
HER_3112	Heron Island	MG385349.1	Keyse et al., 2018
HER_3111	Heron Island	MG385348.1	Keyse et al., 2018
HER_3110	Heron Island	MG385347.1	Keyse et al., 2018
HER_21	Heron Island	MG385346.1	Keyse et al., 2018
ET655	Lizard Island	JX974944.1	Huelsken et al., 2013
ET654	Lizard Island	JX974943.1	Huelsken et al., 2013
ET650	Lizard Island	JX974942.1	Huelsken et al., 2013
ET49	Heron Island	JX974941.1	Huelsken et al., 2013
ET43	Heron Island	JX974940.1	Huelsken et al., 2013
ET375	Lihou Reef	JX974939.1	Huelsken et al., 2013
ET357	Lihou Reef	JX974938.1	Huelsken et al., 2013
ET305	Lihou Reef	JX974937.1	Huelsken et al., 2013
ET303	Lihou Reef	JX974936.1	Huelsken et al., 2013
ET2793	Lizard Island	JX974935.1	Huelsken et al., 2013
ET26	Heron Island	JX974934.1	Huelsken et al., 2013
ET2395	Solomon Islands	JX974933.1	Huelsken et al., 2013
ET2386	Solomon Islands	JX974932.1	Huelsken et al., 2013
ET2348	Solomon Islands	JX974931.1	Huelsken et al., 2013

ET2145	Solomon Islands	JX974930.1	Huelsken et al., 2013
ET2015	Torres Strait	JX974929.1	Huelsken et al., 2013
ET2011	Torres Strait	JX974928.1	Huelsken et al., 2013
ET2006	Torres Strait	JX974927.1	Huelsken et al., 2013
ET1995	Torres Strait	JX974926.1	Huelsken et al., 2013
P100411	South of Philippines	KF446515.1	DeBoer et al., 2014
P072505	South of Philippines	KF446513.1	DeBoer et al., 2014
P069204	South of Philippines	KF446512.1	DeBoer et al., 2014
P069202	South of Philippines	KF446511.1	DeBoer et al., 2014
P066807	South of Philippines	KF446510.1	DeBoer et al., 2014
P066804	South of Philippines	KF446509.1	DeBoer et al., 2014
P027105	South of Philippines	KF446507.1	DeBoer et al., 2014
P026901	South of Philippines	KF446506.1	DeBoer et al., 2014
P026801	South of Philippines	KF446505.1	DeBoer et al., 2014
P026603	South of Philippines	KF446504.1	DeBoer et al., 2014
P022003	North of Philippines	KF446503.1	DeBoer et al., 2014
P021001	North of Philippines	KF446502.1	DeBoer et al., 2014
P020905	North of Philippines	KF446501.1	DeBoer et al., 2014
P020904	North of Philippines	KF446500.1	DeBoer et al., 2014
P020610	North of Philippines	KF446499.1	DeBoer et al., 2014
P020606	North of Philippines	KF446498.1	DeBoer et al., 2014
P014907	North of Philippines	KF446497.1	DeBoer et al., 2014
P014811	North of Philippines	KF446496.1	DeBoer et al., 2014
P014810	North of Philippines	KF446495.1	DeBoer et al., 2014
P014808	North of Philippines	KF446494.1	DeBoer et al., 2014
P014805	North of Philippines	KF446493.1	DeBoer et al., 2014
P014702	North of Philippines	KF446492.1	DeBoer et al., 2014
P013601	North of Philippines	KF446491.1	DeBoer et al., 2014
P008302	North of Philippines	KF446490.1	DeBoer et al., 2014
P014910	North of Philippines	KF446471.1	DeBoer et al., 2014
P038112	South of Philippines	KF446446.1	DeBoer et al., 2014
P020605	North of Philippines	KF446445.1	DeBoer et al., 2014
P020604	North of Philippines	KF446444.1	DeBoer et al., 2014
TmTul76	Tulear	MN068787.1	Fauvelot et al., 2020
TmTul73	Tulear	MN068786.1	Fauvelot et al., 2020
TmTul53	Tulear	MN068785.1	Fauvelot et al., 2020
TmTul46	Tulear	MN068784.1	Fauvelot et al., 2020
TmTul3	Tulear	MN068783.1	Fauvelot et al., 2020
TmTul28	Tulear	MN068782.1	Fauvelot et al., 2020

TmTul2	Tulear	MN068781.1	Fauvelot et al., 2020
TmTul19	Tulear	MN068780.1	Fauvelot et al., 2020
TmTul11	Tulear	MN068779.1	Fauvelot et al., 2020
TmTul10	Tulear	MN068778.1	Fauvelot et al., 2020
TmTul1	Tulear	MN068777.1	Fauvelot et al., 2020
TmRIb2	Reunion Island	MN068768.1	Fauvelot et al., 2020
TmRIb1	Reunion Island	MN068767.1	Fauvelot et al., 2020
TmRI59	Reunion Island	MN068766.1	Fauvelot et al., 2020
TmRI08	Reunion Island	MN068765.1	Fauvelot et al., 2020
TmJu25	Juan de Nova	MN068764.1	Fauvelot et al., 2020
TmJu21	Juan de Nova	MN068763.1	Fauvelot et al., 2020
TmJu19	Juan de Nova	MN068762.1	Fauvelot et al., 2020
TmJu18	Juan de Nova	MN068761.1	Fauvelot et al., 2020
TmJu13	Juan de Nova	MN068760.1	Fauvelot et al., 2020
TmJu10	Juan de Nova	MN068759.1	Fauvelot et al., 2020
MNHN IM-2009-33500	Tulear	MN068750.1	Fauvelot et al., 2020
MNHN IM-2009-33499	Tulear	MN068749.1	Fauvelot et al., 2020
MNHN IM-2009-33498	Tulear	MN068748.1	Fauvelot et al., 2020
MNHN IM-2009-33497	Tulear	MN068747.1	Fauvelot et al., 2020
MNHN IM-2009-33495	Tulear	MN068746.1	Fauvelot et al., 2020
MNHN IM-2009-33490	Tulear	MN068745.1	Fauvelot et al., 2020
MNHN IM-2009-33489	Tulear	MN068744.1	Fauvelot et al., 2020
MNHN IM-2009-33487	Tulear	MN068743.1	Fauvelot et al., 2020
MNHN IM-2009-33485	Tulear	MN068742.1	Fauvelot et al., 2020
MNHN IM-2009-33484	Tulear	MN068741.1	Fauvelot et al., 2020
MNHN IM-2009-33482	Tulear	MN068740.1	Fauvelot et al., 2020
MNHN IM-2009-33480	Tulear	MN068739.1	Fauvelot et al., 2020
MNHN IM-2009-33478	Tulear	MN068738.1	Fauvelot et al., 2020
MNHN IM-2009-33474	Tulear	MN068737.1	Fauvelot et al., 2020
MNHN IM-2009-33472	Tulear	MN068736.1	Fauvelot et al., 2020
MNHN IM-2009-33471	Tulear	MN068735.1	Fauvelot et al., 2020
MNHN IM-2009-33467	Tulear	MN068734.1	Fauvelot et al., 2020
MNHN IM-2009-13077	Tulear	MN068733.1	Fauvelot et al., 2020
MNHN IM-2009-13076	Tulear	MN068732.1	Fauvelot et al., 2020
MNHN IM-2009-13074	Tulear	MN068731.1	Fauvelot et al., 2020
YR_1	Nansha Islands	PQ821125	present study
YR_2	Nansha Islands	PQ821126	present study
YR_3	Nansha Islands	PQ821127	present study
YR_4	Nansha Islands	PQ821128	present study

YR_5	Nansha Islands	PQ821129	present study
YR_6	Nansha Islands	PQ821130	present study
YR_7	Nansha Islands	PQ821131	present study
YR_8	Nansha Islands	PQ821132	present study
YR_9	Nansha Islands	PQ821133	present study
YR_10	Nansha Islands	PQ821134	present study
YR_11	Nansha Islands	PQ821135	present study
YR_12	Nansha Islands	PQ821136	present study
YR_13	Nansha Islands	PQ821137	present study
YR_14	Nansha Islands	PQ821138	present study
YR_15	Nansha Islands	PQ821139	present study
YR_16	Nansha Islands	PQ821140	present study
YR_17	Nansha Islands	PQ821141	present study
YR_18	Nansha Islands	PQ821142	present study
YR_19	Nansha Islands	PQ821143	present study
MR_1	Nansha Islands	PQ821144	present study
MR_2	Nansha Islands	PQ821145	present study
MR_3	Nansha Islands	PQ821146	present study
MR_4	Nansha Islands	PQ821147	present study
MR_5	Nansha Islands	PQ821148	present study
MR_6	Nansha Islands	PQ821149	present study
MR_7	Nansha Islands	PQ821150	present study
MR_8	Nansha Islands	PQ821151	present study
MR_9	Nansha Islands	PQ821152	present study
MR_10	Nansha Islands	PQ821153	present study
MR_11	Nansha Islands	PQ821154	present study
MR_12	Nansha Islands	PQ821155	present study
MR_13	Nansha Islands	PQ821156	present study
MR_14	Nansha Islands	PQ821157	present study
MR_15	Nansha Islands	PQ821158	present study
MR_16	Nansha Islands	PQ821159	present study
tmhap_135	Kenya	HE995487.1	Hui et al., 2016
tmhap_134	Kenya	HE995486.1	Hui et al., 2016
tmhap_133	Kenya	HE995485.1	Hui et al., 2016
tmhap_132	Kenya	HE995484.1	Hui et al., 2016
tmhap_131	Kenya	HE995483.1	Hui et al., 2016
tmhap_130	Kenya	HE995482.1	Hui et al., 2016
tmhap_129	Kenya	HE995481.1	Hui et al., 2016
tmhap_128	French Polynesia	HE995480.1	Hui et al., 2016

tmhap_127	French Polynesia	HE995479.1	Hui et al., 2016
tmhap_126	French Polynesia	HE995478.1	Hui et al., 2016
tmhap_125	French Polynesia	HE995477.1	Hui et al., 2016
tmhap_124	French Polynesia	HE995476.1	Hui et al., 2016
tmhap_123	French Polynesia	HE995475.1	Hui et al., 2016
tmhap_122	Indonesia	HE995474.1	Hui et al., 2016
tmhap_121	Indonesia	HE995473.1	Hui et al., 2016
tmhap_120	Thailand	HE995472.1	Hui et al., 2016
tmhap_119	Thailand	HE995471.1	Hui et al., 2016
tmhap_118	Thailand	HE995470.1	Hui et al., 2016
tmhap_117	Thailand	HE995469.1	Hui et al., 2016
tmhap_116	Thailand	HE995468.1	Hui et al., 2016
tmhap_115	Thailand	HE995467.1	Hui et al., 2016
tmhap_114	Thailand	HE995466.1	Hui et al., 2016
tmhap_113	Thailand	HE995465.1	Hui et al., 2016
tmhap_112	Thailand	HE995464.1	Hui et al., 2016
tmhap_111	Thailand	HE995463.1	Hui et al., 2016
tmhap_110	Thailand	HE995462.1	Hui et al., 2016
tmhap_109	Thailand	HE995461.1	Hui et al., 2016
tmhap_108	Thailand	HE995460.1	Hui et al., 2016
tmhap_107	Thailand	HE995459.1	Hui et al., 2016
tmhap_106	Thailand	HE995458.1	Hui et al., 2016
tmhap_105	Thailand	HE995457.1	Hui et al., 2016
tmhap_104	Thailand	HE995456.1	Hui et al., 2016
tmhap_103	Thailand	HE995455.1	Hui et al., 2016
tmhap_102	Thailand	HE995454.1	Hui et al., 2016
Hap_117	Indonesia	FM244619.1	Nuryanto and Kochzius, 2009
Hap_116	Indonesia	FM244618.1	Nuryanto and Kochzius, 2009
Hap_115	Indonesia	FM244617.1	Nuryanto and Kochzius, 2009
Hap_114	Indonesia	FM244616.1	Nuryanto and Kochzius, 2009
Hap_113	Indonesia	FM244615.1	Nuryanto and Kochzius, 2009
Hap_112	Indonesia	FM244614.1	Nuryanto and Kochzius, 2009
Hap_111	Indonesia	FM244613.1	Nuryanto and Kochzius, 2009
Hap_110	Indonesia	FM244612.1	Nuryanto and Kochzius, 2009
Hap_109	Indonesia	FM244611.1	Nuryanto and Kochzius, 2009
Hap_108	Indonesia	FM244610.1	Nuryanto and Kochzius, 2009
Hap_107	Indonesia	FM244609.1	Nuryanto and Kochzius, 2009
Hap_106	Indonesia	FM244608.1	Nuryanto and Kochzius, 2009
Hap_105	Indonesia	FM244607.1	Nuryanto and Kochzius, 2009

Hap_104	Indonesia	FM244606.1	Nuryanto and Kochzius, 2009
Hap_103	Indonesia	FM244605.1	Nuryanto and Kochzius, 2009
Hap_102	Indonesia	FM244604.1	Nuryanto and Kochzius, 2009
Hap_101	Indonesia	FM244603.1	Nuryanto and Kochzius, 2009
Hap_100	Indonesia	FM244602.1	Nuryanto and Kochzius, 2009
Hap_99	Indonesia	FM244601.1	Nuryanto and Kochzius, 2009
Hap_98	Indonesia	FM244600.1	Nuryanto and Kochzius, 2009
Hap_97	Indonesia	FM244599.1	Nuryanto and Kochzius, 2009
Hap_96	Indonesia	FM244598.1	Nuryanto and Kochzius, 2009
Hap_95	Indonesia	FM244597.1	Nuryanto and Kochzius, 2009
Hap_94	Indonesia	FM244596.1	Nuryanto and Kochzius, 2009
Hap_93	Indonesia	FM244595.1	Nuryanto and Kochzius, 2009
Hap_92	Indonesia	FM244594.1	Nuryanto and Kochzius, 2009
Hap_91	Indonesia	FM244593.1	Nuryanto and Kochzius, 2009
Hap_90	Indonesia	FM244592.1	Nuryanto and Kochzius, 2009
Hap_89	Indonesia	FM244591.1	Nuryanto and Kochzius, 2009
Hap_88	Indonesia	FM244590.1	Nuryanto and Kochzius, 2009
Hap_87	Indonesia	FM244589.1	Nuryanto and Kochzius, 2009
Hap_86	Indonesia	FM244588.1	Nuryanto and Kochzius, 2009
Hap_85	Indonesia	FM244587.1	Nuryanto and Kochzius, 2009
Hap_84	Indonesia	FM244586.1	Nuryanto and Kochzius, 2009
Hap_83	Indonesia	FM244585.1	Nuryanto and Kochzius, 2009
Hap_82	Indonesia	FM244584.1	Nuryanto and Kochzius, 2009
Hap_81	Indonesia	FM244583.1	Nuryanto and Kochzius, 2009
Hap_80	Indonesia	FM244582.1	Nuryanto and Kochzius, 2009
Hap_79	Indonesia	FM244581.1	Nuryanto and Kochzius, 2009
Hap_78	Indonesia	FM244580.1	Nuryanto and Kochzius, 2009
Hap_77	Indonesia	FM244579.1	Nuryanto and Kochzius, 2009
Hap_76	Indonesia	FM244578.1	Nuryanto and Kochzius, 2009
Hap_75	Indonesia	FM244577.1	Nuryanto and Kochzius, 2009
Hap_74	Indonesia	FM244576.1	Nuryanto and Kochzius, 2009
Hap_73	Indonesia	FM244575.1	Nuryanto and Kochzius, 2009
Hap_72	Indonesia	FM244574.1	Nuryanto and Kochzius, 2009
Hap_71	Indonesia	FM244573.1	Nuryanto and Kochzius, 2009
Hap_70	Indonesia	FM244572.1	Nuryanto and Kochzius, 2009
Hap_69	Indonesia	FM244571.1	Nuryanto and Kochzius, 2009
Hap_68	Indonesia	FM244570.1	Nuryanto and Kochzius, 2009
Hap_67	Indonesia	FM244569.1	Nuryanto and Kochzius, 2009
Hap_66	Indonesia	FM244568.1	Nuryanto and Kochzius, 2009

Hap_65	Indonesia	FM244567.1	Nuryanto and Kochzius, 2009
Hap_64	Indonesia	FM244566.1	Nuryanto and Kochzius, 2009
Hap_63	Indonesia	FM244565.1	Nuryanto and Kochzius, 2009
Hap_62	Indonesia	FM244564.1	Nuryanto and Kochzius, 2009
Hap_61	Indonesia	FM244563.1	Nuryanto and Kochzius, 2009
Hap_60	Indonesia	FM244562.1	Nuryanto and Kochzius, 2009
Hap_59	Indonesia	FM244561.1	Nuryanto and Kochzius, 2009
Hap_58	Indonesia	FM244560.1	Nuryanto and Kochzius, 2009
Hap_57	Indonesia	FM244559.1	Nuryanto and Kochzius, 2009
Hap_56	Indonesia	FM244558.1	Nuryanto and Kochzius, 2009
Hap_55	Indonesia	FM244557.1	Nuryanto and Kochzius, 2009
Hap_54	Indonesia	FM244556.1	Nuryanto and Kochzius, 2009
Hap_53	Indonesia	FM244555.1	Nuryanto and Kochzius, 2009
Hap_52	Indonesia	FM244554.1	Nuryanto and Kochzius, 2009
Hap_51	Indonesia	FM244553.1	Nuryanto and Kochzius, 2009
Hap_50	Indonesia	FM244552.1	Nuryanto and Kochzius, 2009
Hap_49	Indonesia	FM244551.1	Nuryanto and Kochzius, 2009
Hap_48	Indonesia	FM244550.1	Nuryanto and Kochzius, 2009
Hap_47	Indonesia	FM244549.1	Nuryanto and Kochzius, 2009
Hap_46	Indonesia	FM244548.1	Nuryanto and Kochzius, 2009
Hap_45	Indonesia	FM244547.1	Nuryanto and Kochzius, 2009
Hap_44	Indonesia	FM244546.1	Nuryanto and Kochzius, 2009
Hap_43	Indonesia	FM244545.1	Nuryanto and Kochzius, 2009
Hap_42	Indonesia	FM244544.1	Nuryanto and Kochzius, 2009
Hap_41	Indonesia	FM244543.1	Nuryanto and Kochzius, 2009
Hap_40	Indonesia	FM244542.1	Nuryanto and Kochzius, 2009
Hap_39	Indonesia	FM244541.1	Nuryanto and Kochzius, 2009
Hap_38	Indonesia	FM244540.1	Nuryanto and Kochzius, 2009
Hap_37	Indonesia	FM244539.1	Nuryanto and Kochzius, 2009
Hap_36	Indonesia	FM244538.1	Nuryanto and Kochzius, 2009
Hap_35	Indonesia	FM244537.1	Nuryanto and Kochzius, 2009
Hap_34	Indonesia	FM244536.1	Nuryanto and Kochzius, 2009
Hap_33	Indonesia	FM244535.1	Nuryanto and Kochzius, 2009
Hap_32	Indonesia	FM244534.1	Nuryanto and Kochzius, 2009
Hap_31	Indonesia	FM244533.1	Nuryanto and Kochzius, 2009
Hap_30	Indonesia	FM244532.1	Nuryanto and Kochzius, 2009
Hap_29	Indonesia	FM244531.1	Nuryanto and Kochzius, 2009
Hap_28	Indonesia	FM244530.1	Nuryanto and Kochzius, 2009
Hap_27	Indonesia	FM244529.1	Nuryanto and Kochzius, 2009

Hap_26	Indonesia	FM244528.1	Nuryanto and Kochzius, 2009
Hap_25	Indonesia	FM244527.1	Nuryanto and Kochzius, 2009
Hap_24	Indonesia	FM244526.1	Nuryanto and Kochzius, 2009
Hap_23	Indonesia	FM244525.1	Nuryanto and Kochzius, 2009
Hap_22	Indonesia	FM244524.1	Nuryanto and Kochzius, 2009
Hap_21	Indonesia	FM244523.1	Nuryanto and Kochzius, 2009
Hap_20	Indonesia	FM244522.1	Nuryanto and Kochzius, 2009
Hap_19	Indonesia	FM244521.1	Nuryanto and Kochzius, 2009
Hap_18	Indonesia	FM244520.1	Nuryanto and Kochzius, 2009
Hap_17	Indonesia	FM244519.1	Nuryanto and Kochzius, 2009
Hap_16	Indonesia	FM244518.1	Nuryanto and Kochzius, 2009
Hap_15	Indonesia	FM244517.1	Nuryanto and Kochzius, 2009
Hap_14	Indonesia	FM244516.1	Nuryanto and Kochzius, 2009
Hap_13	Indonesia	FM244515.1	Nuryanto and Kochzius, 2009
Hap_12	Indonesia	FM244514.1	Nuryanto and Kochzius, 2009
Hap_11	Indonesia	FM244513.1	Nuryanto and Kochzius, 2009
Hap_10	Red Sea	FM244485.1	Nuryanto and Kochzius, 2009
Hap_9	Red Sea	FM244484.1	Nuryanto and Kochzius, 2009
Hap_8	Red Sea	FM244483.1	Nuryanto and Kochzius, 2009
Hap_7	Red Sea	FM244482.1	Nuryanto and Kochzius, 2009
Hap_6	Red Sea	FM244481.1	Nuryanto and Kochzius, 2009
Hap_5	Red Sea	FM244480.1	Nuryanto and Kochzius, 2009
Hap_4	Red Sea	FM244479.1	Nuryanto and Kochzius, 2009
Hap_3	Red Sea	FM244478.1	Nuryanto and Kochzius, 2009
Hap_2	Red Sea	FM244477.1	Nuryanto and Kochzius, 2009
Hap_1	Red Sea	FM244476.1	Nuryanto and Kochzius, 2009
